# Cytokinesis-block micronucleus assay of celecoxib and celecoxib derivatives

**DOI:** 10.1016/j.toxrep.2020.11.004

**Published:** 2020-11-20

**Authors:** Hauke Reimann, Quoc Anh Ngo, Helga Stopper, Henning Hintzsche

**Affiliations:** a Institute of Pharmacology and Toxicology, University of Würzburg, Versbacher Straße 9, 97078 Würzburg, Germany; b Institute of Chemistry, Vietnam Academy of Science and Technology, 18 Hoang Quoc Viet, CauGiay, Hanoi, Viet Nam; c Bavarian Health and Food Safety Authority, Eggenreuther Weg 43, 91058 Erlangen, Germany

**Keywords:** Celecoxib, DNA damage, Micronucleus test, Chemoprevention

## Abstract

Celecoxib is used widely for the acute treatment of pain and for pain relief in various diseases. Furthermore, it shows potential in chemoprevention, although chronic treatment with celecoxib could lead to adverse effects like cardiovascular events. New derivatives of celecoxib were synthesised that may be suitable as chemopreventive agent without inducing adverse effects.

Critical endpoint for a safe use of pharmaceuticals is genotoxicity after application. A standard test for the assessment of genotoxicity is the cytokinesis-block micronucleus assay, that evaluates the number micronuclei after treatment of cells with a test compound as biomarker for DNA damage. Various promising derivatives of celecoxib have been assessed with the cytokinesis-block micronucleus assay in HeLa-H2B-GFP cells. It could be demonstrated, that neither celecoxib nor its derivatives were genotoxic in this assay and therefore celecoxib derivatives could be developed further for a safe use as chemopreventive agent.

## Highlights

• New derivatives of celecoxib can improve beneficial effects with better safety profile.
• DNA damage in form of micronuclei has not been observed after treatment with celecoxib or any derivative.
• Further development of celecoxib derivatives for chemoprevention may be promising.

## 1 Introduction

The non-steroidal anti-inflammatory drug (NSAID) celecoxib has numerous indications from the treatment of acute pain to a broad range of other analgesic therapies e.g. in osteoarthritis or rheumatoid arthritis [[1], [2], [3]]. Celecoxib is a selective inhibitor of the cyclooxygenase-2 (COX-2), which plays a major role in the synthesis of prostaglandins [4]. Besides its main use in pain relief, celecoxib has also shown potential as chemopreventive agent for the treatment of breast, lung or other cancer forms [5]. It was found, that COX-2 inhibition and tumour growth suppression were associated with different structural regions of the molecule and therefore derivatives could in theory still inhibit tumour growth without affecting COX2 [6]. This could be beneficial, as long-term treatment with COX-2 inhibitors may result in an increased risk of cardiovascular events [7].

Recently, several derivatives of celecoxib were synthesised and their biological activity assessed via cytotoxicity testing in three different human cancer cell lines. Furthermore, the NO inhibition as a marker for potential cardiovascular risk was measured. Some of these new derivatives showed a stronger inhibition of cell growth in some or all three different human cancer cell lines while NO inhibition was similar or reduced compared to celecoxib [8]. This could be an interesting starting point for developing new therapeutics, as an increased therapeutic index could lead to a safer and more effective treatment of some cancer types. Other analogues or derivatives of celecoxib showed an increased COX-2 inhibition and higher selectivity, which shows the potential of novel chemical structures to improve beneficial effects of celecoxib [9].

Besides cardiovascular effects, genotoxicity is a critical endpoint for a safe use of pharmaceuticals. An important assay to evaluate genotoxicity is the cytokinesis-block micronucleus test, which is widely used in routine testing for chemicals, pharmaceuticals or other substances [10,11]. Micronuclei are small nuclear compartments comprising chromosomal fragments or whole chromosomes [12]. They are formed due to mis-attachment or disturbances of microtubules or double strand breaks caused from mis-repair or breakage of chromatin bridges [13]. The consequences of micronuclei for cell proliferation are still not fully understood, especially the relevance of micronuclei for growth, senescence or death of cells after several cell cycles [14]. Recently, micronuclei have been shown to be a central mechanism for the introduction of massive rearrangements in single chromosomes, leading to chromothripsis, that may cause rapid tumour evolution and be the reason for heterogeneity of tumour cells [15,16].

Besides the use as a biomarker in genotoxicity testing, micronuclei in human blood lymphocytes are predictive for cancer risk, which makes them a suitable tool to assess the effect of lifestyle factors or diseases on the risk of tumour formation [17]. Furthermore, analysing micronucleus frequency could be a promising tool for monitoring tumours like colorectal cancer [18]. Micronuclei can be evaluated not only in blood lymphocytes, but also in various other tissues like buccal cells or cultivated cells in vitro [11,19]. In this study, we performed the micronucleus test after treatment of HeLa cells with celecoxib and four different derivatives to assess the putative potential of these substances to induce DNA damage.

## 2 Materials and methods

### 2.1 Cell culture and treatment conditions

H2B-GFP-HeLa cells were used for all experiments (provided by Noriaki Shimizu, Graduate School of Integrated Sciences for Life, Hiroshima University, Japan) [20]. Cells were cultivated at 37 °C and 5% CO_2_ in DMEM High Glucose (Sigma-Aldrich) without phenol red but supplemented with 10 % FCS (Merck), 2 mM L-glutamine (Sigma), 100 μg/mL streptomycin (Sigma), 100 U/mL penicillin (Sigma), 1 mM sodium pyruvate (Sigma) and 25 mM HEPES (Sigma). Etoposide was supplied by Teva, all other test materials were synthesised as described before [8]. Treatment duration was 3 h for etoposide and 4 h for all other test compounds. All test compounds were synthesised according to methods described previously [8]. Solvent for all substances was dimethyl sulfoxide (DMSO), which was also used in a quantity of 1% as negative control.

### 2.2 Cytokinesis-block micronucleus test

After treatment, 3 μg/mL cytochalasin B was added and cells were cultivated for additional 22−24 hours. Next, cytospinning was performed onto slides, and the preparations were then fixed for at least 2 h in −20 °C methanol before staining with GelGreen (Biotium). Micronucleus frequency analysis in binucleated cells was performed as described before [10]. Mono-, bi- and multinucleated cells as well as mitotic and apoptotic cells were counted in 1000 cells, while micronuclei were counted in 1000 binucleated cells. Cytokinesis-block proliferation index (CBPI) was calculated with the following formula:

$$\frac{1\text{*}\left(No.\;of\;mononuclear\;cells\right)+2\text{*}\left(No.\;of\;binuclear\;cells\right)+3\text{*}(No.\;of\;multinuclear\;cells)}{\left(No.\;of\;mononuclear\;cells\right)+\left(No.\;of\;binuclear\;cells\right)+(No.\;of\;multinuclear\;cells)}$$

Each scoring was conducted in three independent experiments on two slides each. For statistical analysis, Mann-Whitney-*U*-test was conducted and significance assumed when p ≤ 0.05.

## 3 Results and discussion

**Fig. 1:**
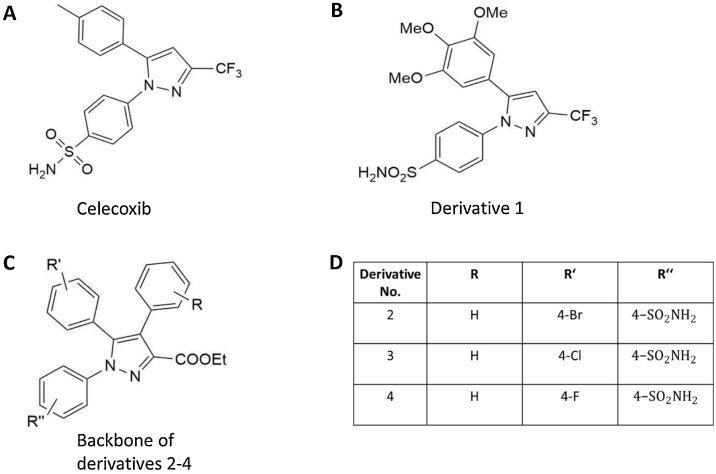
Test materials used for genotoxicity testing. A) Structure of celecoxib. B) Structure of the 3,4,5-trimethoxyphenyl analogue of celecoxib. C) Structure of ethyl 1,4,5-triaryl-1H-pyrazole-3-carboxylate. D) Structure of derivative 2-4.

The structure of all test materials are provided in Fig. 1. Celecoxib has a diaryl-substituted pyrazole structure (Fig. 1a), while derivative 1 is a 3,4,5-trimethoxyphenyl analogue of celecoxib (Fig. 1b). Derivative 2–4 are all based on the same ethyl 1,4,5-triaryl-1*H*-pyrazole-3-carboxylate structure (Fig. 1c) with different substituents at positions R, R’ and R’’ (Fig. 1d).

**Fig. 2:**
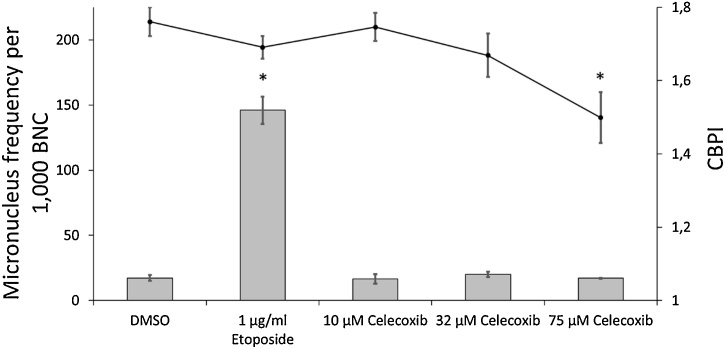
Micronucleus test of celecoxib. Micronucleus frequency (grey bar) per 1000 binucleated cells (BNC) and cytokinesis-block proliferation index (CBPI, black line). Mean of three independent experiments ± standard error. Asterisk represent p ≤ 0.05 compared to treatment with 1% DMSO (Mann-Whitney-*U*-test).

**Fig. 3:**
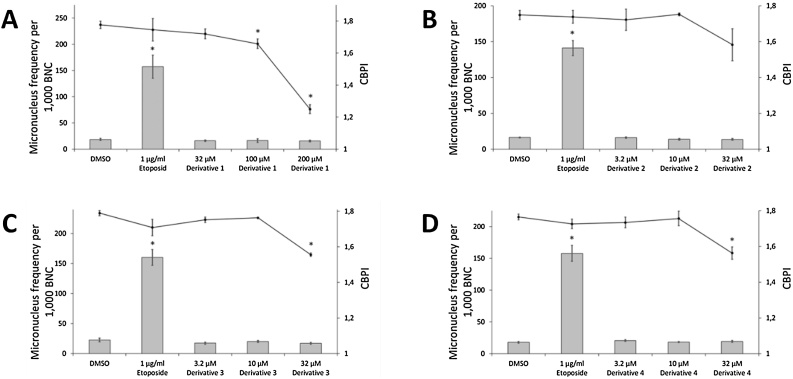
Micronucleus test of celecoxib derivatives 1-4 (A-D). Micronucleus frequency (grey bar) per 1000 binucleated cells (BNC) and cytokinesis-block proliferation index (CBPI, black line) were shown. Mean of three independent experiments ± standard error. Asterisk represent p ≤ 0.05 compared to treatment with 1% DMSO (Mann-Whitney-*U*-test).

Micronucleus tests of celecoxib and all derivatives were performed. Celecoxib showed no induction of micronuclei up to 75 μM when compared to negative control (Fig. 2). After treatment with 100 μM most cells were not viable, which made an evaluation impossible (data not shown for all tested non-viable conditions). Derivative 1 did not increase micronucleus frequency up to 200 μM (Fig. 3a). Proliferation was clearly decreased at 200 μM, while at 300 μM most cells were not viable making evaluation impossible. Micronucleus induction was also not observed for derivatives 2–4, but they all showed a clear decrease of proliferation already at 32 μM, which was significant for derivative 3 and 4 (Fig. 3b-d). 100 μM treatment was cytotoxic for derivatives 2–4, which all share the same ethyl 1,4,5-triaryl-1*H*-pyrazole-3-carboxylate structure.

Taken together, these data demonstrate that neither celecoxib nor its derivatives showed an induction of micronuclei in HeLa cells. This is in line with former studies for celecoxib showing no carcinogenicity in rats up to doses of 200 mg/kg daily for two years and no mutagenicity or clastogenicity in the Ames test, in the chromosomal aberration assay in Chinese Hamster ovary cells, and in the in vivo micronucleus test in rat bone marrow [21]. Incubation of whole blood with celecoxib even reduced the induction of micronuclei caused by ionising radiation in a cytokinesis-block micronucleus test in human lymphocytes [22]. In contrast, another study demonstrated a more complicated relation between celecoxib and DNA damage: While accumulation of DNA-adducts was observed in lung and heart of smoke-free mice after exposure to celecoxib, smoke-induced DNA-damage was reduced after treatment with celecoxib, that made the authors propose multiple mechanisms for celecoxib to interact with DNA [23].

Celecoxib was suggested as chemopreventive agent [24]. For this application, side effects like cardiovascular events have to be considered, but also gastrointestinal adverse effects like gastroduodenal ulcerations may occur after prolonged application, although celecoxib showed a lower incidence than diclofenac or ibuprofen [25]. Various types of substances can be used for this purpose from hormonal agents to vaccines, but also different NSAIDs showed chemopreventive effects when applied for several years [26]. Prostaglandins might play an important role in chemoprevention, as they can be produced by tumour cells and stimulate tumour growth depending on the activity of COX-2. COX-2 is considered to be a main target for chemopreventive action, therefore selective COX-2 inhibitors like celecoxib are promising substances, as their selectivity reduces the probability of adverse effects related to COX-1 inhibition [27]. Celecoxib is under investigation for chemoprevention of colorectal adenomas and long-term studies showed a clear reduction of recurrent adenomas – but higher doses (400 mg twice daily) pose the risk of severe cardiovascular effects making correct dosing crucial for the outcome of the treatment [24]. In addition, celecoxib could be beneficial in radiotherapy by preventing normal tissue to get damaged and sensitise tumour cells to radiation [28]. Moreover, celecoxib may also be used in cutaneous formulation e.g. in chemoprevention of skin cancer as a more effective delivery system with reduced systemic toxicity [29].

New derivatives may improve beneficial effects of celecoxib while reducing its toxicity [8]. Genotoxicity in HeLa cells was not observed in the tested concentrations, and higher doses only lead to excessive cytotoxicity, which made evaluation impossible. However, it remains unclear, if there is any DNA damage, when other cell lines or endpoints like mutation rate in bacteria or double strand breaks are considered. Furthermore, bioactivation of celecoxib and celecoxib derivatives could potentially lead to genotoxic metabolites which is an important open question, that should be investigated in future studies, e.g. by using a metabolically active cell line like HepG2 cells or bioactivation by S9 mix. Effects of mixtures of chemicals may show different effects than substances alone, even at very low doses [30,31]. As this may also occur when celecoxib is administered along with other pharmaceuticals, a comprehensive genotoxicity assessment should also consider combination effects. Although more information on the pharmacological activity of the derivatives is necessary, the limited data presently available suggest promising potential for these compounds.

## Funding

This publication was supported by the Open Access Publication Fund of the University of Würzburg.

## CRediT authorship contribution statement

**Hauke Reimann:** Writing - original draft, Investigation. **Quoc Anh Ngo:** Writing - review & editing, Investigation, Resources. **Helga Stopper:** Writing - review & editing, Validation. **Henning Hintzsche:** Conceptualization, Writing - original draft, Supervision.

## Declaration of Competing Interest

The authors report no declarations of interest.
